# Effect of excess weight and insulin resistance on DNA methylation in prepubertal children

**DOI:** 10.1038/s41598-022-12325-y

**Published:** 2022-05-19

**Authors:** Pedro Barbosa, Reid D. Landes, Stefan Graw, Stephanie D. Byrum, Sirish Bennuri, Leanna Delhey, Chris Randolph, Stewart MacLeod, Andreia Reis, Elisabet Børsheim, Shannon Rose, Eugenia Carvalho

**Affiliations:** 1 PhD Programme in Experimental Biology and Biomedicine, Institute for Interdisciplinary Research (IIIUC), University of Coimbra, Coimbra, Portugal; 2 Center for Neuroscience and Cell Biology, University of Coimbra, Coimbra, Portugal; 3 Institute for Interdisciplinary Research (IIIUC), University of Coimbra, Coimbra, Portugal; 4 Department of Biostatistics, University of Arkansas for Medical Sciences, Little Rock, AR USA; 5 Department of Biochemistry and Molecular Biology, University of Arkansas for Medical Sciences, Little Rock, AR USA; 6 Arkansas Children’s Research Institute, Little Rock, AR USA; 7 Everest Clinical Research Corporation, Markham, ON Canada; 8 Department of Epidemiology, University of Arkansas for Medical Sciences, Little Rock, AR USA; 9 Department of Medical Sciences (DCM), Institute for Research in Biomedicine (iBiMED), University of Aveiro, Aveiro, Portugal; 10 Department of Pediatrics, University of Arkansas for Medical Sciences, Little Rock, AR USA; 11 Arkansas Children’s Nutrition Center, Little Rock, AR USA; 12 Department of Geriatrics, University of Arkansas for Medical Sciences, Little Rock, AR USA

**Keywords:** Paediatric research, Computational biology and bioinformatics, Physiology, Endocrinology

## Abstract

Epigenetic mechanisms, such as DNA methylation, regulate gene expression and play a role in the development of insulin resistance. This study evaluates how the BMI *z*-score (BMIz) and the homeostatic model assessment of insulin resistance (HOMA-IR), alone or in combination, relate to clinical outcomes and DNA methylation patterns in prepubertal children. DNA methylation in peripheral blood mononuclear cells (PBMCs) and clinical outcomes were measured in a cohort of 41 prepubertal children. Children with higher HOMA-IR had higher blood pressure and plasma lactate levels while children with higher BMIz had higher triglycerides levels. Moreover, the DNA methylation analysis demonstrated that a 1 unit increase in the BMIz was associated with a 0.41 (95% CI: 0.29, 0.53) increase in methylation of a CpG near the *PPP6R2* gene. This gene is important in the regulation of NF-kB expression. However, there was no strong evidence that the BMIz and the HOMA-IR were synergistically related to any clinical or DNA methylation outcomes. In summary, the results suggest that obesity and insulin resistance may impact metabolic health both independently in prepubertal children. In addition, obesity also has an impact on the DNA methylation of the *PPP6R2* gene. This may be a novel underlying starting point for the systemic inflammation associated with obesity and insulin resistance, in this population.

## Introduction

Overweight and obesity are a growing public health concern^1^. Excess weight affects the adult population, but also an increasing number of children, with about 43 million preschool children worldwide affected in 2010^2^. Between 2017–2018, ~ 16.1% of children and adolescents (2–19 years) in the USA were overweight while ~ 19.3% were obese^3^. Excessive weight gain in early childhood and in adolescence is associated with impaired insulin action^4^ and the development of comorbidities, such as cardiometabolic diseases^5^, cancer^6,7^, hypertension^8^ and type 2 diabetes (T2D)^7,9^, later in life. Being overweight or obese early in life also increases the odds of acquiring comorbidities at a younger age, such as, cardiovascular disease, T2D and non-alcoholic fatty liver disease^10^.

One of the major links between chronic disease development and overweight or obesity is insulin resistance (IR)^11–13^. IR is characterized by impaired insulin action and consequently a reduction in glucose uptake by insulin sensitive tissues, including skeletal muscle, liver and adipose tissue^4^. This condition can arise and be present many years before T2D is diagnosed, even in a setting of normal fasting glycemia^11,14^. Euglycemia, in this case, is maintained by an increased production of insulin by pancreatic β-cells^15^. Interestingly, not all subjects with obesity develop IR as some people appear to be protected and remain metabolically healthy^16–18^.

The reason why some people develop IR while others not, cannot be described exclusively by genetic variability^17–19^. More recently, epigenetic modifications have been identified as playing a crucial role in metabolic disease development^20,21^, including obesity and IR^6,19,22^. These epigenetic modifications may serve as adaptive mechanisms of the genome toward changes in lifestyle and environmental factors^6^.

Different epigenetic mechanisms regulate gene expression. These include histone methylation, histone acetylation, microRNA-regulation and DNA methylation (DNAm)^23,24^. DNAm is one of the best described epigenetic markers^19^. DNAm can be modified by lifestyle including negative behaviors, such as smoking and poor nutrition, as well as environmental exposures, including air pollution^25^. Modifications in DNAm have already been described to occur even in-utero^26^. These are also important for the development and differentiation of tissues during development^27^.

It is important to identify and understand important epigenetic modifications that may occur at an early stage, in children with obesity, to facilitate prevention and treatments of future comorbidities associated with obesity and IR. The aim of this study was to evaluate how BMIz and IR alone or in combination were related with clinical parameters, such as blood pressure, the lipid profile and lactate levels, as well as DNAm patterns in genome-wide analyses of PBMCs, obtained from prepubertal children 5–10 years of age.

## Methods

### Study participants

After approval by the Institutional Review Board (IRB) at the University of Arkansas for Medical Science and following the Declaration of Helsinki (1964) guidelines, a cohort of 42 prepubertal children with ages between 5 and 9 years old was recruited for the present study. The recruitment was performed considering the following inclusion criteria—ages 5 to 9 years (at the date of the visit)—and exclusion criteria—being classified as underweight accordingly to the CDC growth charts (https://www.cdc.gov/growthcharts); the presence of chronic conditions/diseases such as type 1 diabetes and autoimmune diseases or the use of medication that could affect the study outcomes. The study is registered in the ClinicalTrials.gov, with reference NCT03323294. The parents or legal guardians of the study participants gave written informed consent, and children older than 7 years of age gave assent to participate. During the data analysis, participants with age ≥ 9 years and 6 months but < 10 years, were considered 10 years old.

After arrival to Pediatric Clinical Research Unit (PCRU) at Arkansas Children’s Hospital, after an overnight fast, a calibrated Avery Berkel HL122 Series Platform Scale (Dynamic Scales, Terre Haute, IN, USA) was used to measure the weight, while height was registered using a stadiometer (Novel Products, Rockton, IL, USA). To measure the heart rate (bpm), as well as systolic and diastolic blood pressure (mmHg), a GE Carescape V100 Dinamap vital signs monitor was used following the standard procedure at PCRU. Furthermore, adjusting for height, age, and sex, we computed the percentiles of systolic and diastolic blood pressure using the calculator from the Canadian Pediatric Endocrine Group (https://cpeg-gcep.shinyapps.io/BPz_cpeg/) and then converted the percentiles into *z*-scores using the R function *qnorm* for each participant. Blood samples were collected and processed as previously^28^. Plasma was aliquoted and replaced using PBS supplemented with 2 mM EDTA and 0.1% BSA (Sigma Aldrich, St. Louis, MO). Thereafter, the PBMCs were isolated using a gradient separation, counted and snap frozen. Both, plasma and PBMCs were stored up to 1–2 years at −80 °C until the study conclusion.

Fasting plasma glucose was quantified using a YSI 2900 biochemistry analyzer (YSI Life Sciences, Yellow Springs, OH, USA), while fasting plasma insulin (µIU/mL) was measured resorting to the Mesoscale Discovery Platform (MSD Multi-Array Assay System, Gaithersburg, MD, USA). To quantify the lipid profile—high density lipoprotein (HDL; mmol/L), low density lipoprotein (LDL; mmol/L), triglycerides (mmol/L), non-esterified fatty acids (NEFA; mmol/L) and glycerol (µmol/L)—an RX Daytona clinical analyzer was used following the manufacturer’s instructions (Randox Laboratories-IS limited, Kearneysville, WV, USA). Furthermore, plasma lactate concentration was measured using the same technology.

From previous studies, about 30% of subjects with obesity have IR. Therefore, study participants were stratified based on their BMI and IR levels^17,18^. Following the guidelines of the Center for Diseases Control and Prevention (https://www.cdc.gov/growthcharts), the BMI *z*-score (BMIz) was calculated using the calculator from the Canadian Pediatric Endocrine Group (https://cpeg-gcep.shinyapps.io/quickZ_CDC/). The study participants were classified as normal weight when their age- and sex-adjusted BMI was below the 85^th^ percentile, or were also classified as overweight, which in this study also included subjects with obesity, when their age- and sex-adjusted BMI was above the 85th percentile (i.e., BMIz > 1.036). Furthermore, participants were classified as IR when their HOMA-IR was greater than 2.0^29^. The HOMA index was calculated as previously described by Matthews, et al.^30^ Combinations of these two classifications (BMIz and HOMA-IR) produced three groups of participants: Normal weight and insulin sensitive (N-IS), overweight and insulin sensitive (O-IS), and overweight and insulin resistant (O-IR). Summary statistics and results for the three groups separately and overall are presented in Table 1.

**Table 1:** Summary statistics of characteristics and outcomes from the study participants, overall and categorized by weight and the insulin resistance status.

Characteristic	Overall (*n* = 41)	Normal weight-insulin sensitive (*n* = 14)	Overweight-insulin sensitive (*n* = 16)	Overweight-insulin resistant (*n* = 11)
Non-Hispanic Caucasian	51%	64%	63%	18%
Male	56%	71%	44%	55%
Age (years)	7.5 ± 1.3	7.0 ± 1.2	7.6 ± 1.3	7.9 ± 1.2
Weight (kg)	36.1 ± 12.4	24.8 ± 4.7	36.9 ± 9.1	49.4 ± 9.4
Height (cm)	130.6 ± 10.1	124.7 ± 9.1	131.2 ± 9.8	137.3 ± 7.5
BMI	20.6 ± 4.7	15.8 ± 1.0	21.1 ± 2.9	26.1 ± 3.3
BMI *z*-score	1.37 ± 1.04	0.15 ± 0.60	1.76 ± 0.46	2.36 ± 0.38
Glucose (mmol/L)	4.91 ± 0.48	4.88 ± 0.39	4.78 ± 0.47	5.12 ± 0.58
Insulin (µU/mL)	8.88 ± 8.50	3.77 ± 1.40	5.79 ± 2.37	19.89 ± 9.62
HOMA-IR	1.99 ± 2.07	0.82 ± 0.31	1.21 ± 0.46	4.60 ± 2.50
**Modeled clinical outcomes**^a,b^				
Heart rate (bpm)	75.2 (68.5, 81.9)	76.6 (71.3, 82.0)^A,B^	73.5 (69.3, 77.8)^A^	81.9 (75.1, 88.8)^B^
Diastolic blood pressure *z*-score	0.34 (−0.12, 0.80)	−0.05 (−0.45, 0.35)^A^	0.23 (−0.09, 0.55)^A^	0.81 (0.34, 1.29)^B^
Systolic blood pressure *z*-score	1.01 (0.55, 1.48)	0.29 (−0.11, 0.70)^A^	0.83 (0.50, 1.15)^A^	1.52 (1.04, 2.00)^B^
Low density lipoprotein (mmol/L)	2.30 (1.81, 2.79)	2.13 (1.74, 2.52)^A^	2.32 (2.01, 2.62)^A^	2.72 (2.22, 3.31)^A^
High density lipoprotein (mmol/L)^d^	1.34 (1.16, 1.52)	1.45 (1.30, 1.59)^A^	1.36 (1.24, 1.47)^A^	1.16 (0.98, 1.34)^A^
Triglycerides (mmol/L)	0.76 (0.51, 1.02)	0.50 (0.30, 0.70)^A^	0.82 (0.66, 0.98)^B^	0.97 (0.71, 1.22)^B^
Total cholesterol (mmol/L)	3.71 (3.18, 4.24)	3.77 (3.35, 4.19) ^A^	3.8 (3.47, 4.14) ^A^	3.98 (3.44, 4.51) ^A^
Glycerol (µmol/L)	75.7 (53.4, 98)	85.9 (68.2, 103.6)^A^	92.3 (78.3, 106.2)^A^	88.4 (65.7, 111)^A^
Lactate (mmol/L)	2.29 (1.95, 2.62)	1.94 (1.67, 2.21)^A^	2.06 (1.85, 2.27)^A^	2.67 (2.33, 3.02)^B^
NEFA^c^ (mmol/L)	0.08 (0.05, 0.11)	0.10 (0.07, 0.12)^A^	0.09 (0.08, 0.11)^A^	0.07 (0.04, 0.1)^A^

Characteristics are presented as percentages of *N* (or *n*) or as means ± standard deviations. Clinical outcomes are presented as means and 95% confidence intervals.

^a^Means (95% CIs) were estimated from the regression model, and assumed 51% white/non-Hispanic, 56% male, and 7.5 years of age for all groups including Overall; the assumed BMI *z*-score and HOMA-IR were the means for the respective groups.

^b^Groups sharing the same letter (A or B) were not different at the 0.05/3 = 0.017 significance level.

^c^NEFA—Non-esterified fatty acids.

^d^When including the extreme participant, the O-IR mean of high-density lipids was significantly lower than the N-IS mean.

### DNA methylation profiling and data pre-processing

Genome-wide DNA methylation in PBMCs was assessed by the bisulfite-converted genomic DNA after extraction (Puregene Blood Kit; Gentra Systems, Inc., Minneapolis, MN, USA), using the Illumina Infinium Methylation EPIC Bead Chip array (Illumina, San Diego, CA, USA)^31^. This technology interrogates over 850,000 methylation sites covering 99% of the RefSeq (NCBI Reference Sequence Database) genes, 96% of CpG islands (CGIs) with a coverage across promoters, 5' and 3'-UTRs, first exons and gene bodies^31^. Genomic DNA was extracted using the Puregene Blood Kit (Gentra Systems, Inc., Minneapolis, MN, USA), and 500 ng was thereafter bisulfite treated and purified using the EZ DNA Methylation-Gold kit (Zymo Research, Irvine, CA) according to the manufacturer’s protocol^31^. The resultant bisulfite-converted DNA was processed and hybridized to the Illumina Infinium Methylation EPIC Bead Chip. Subsequently, it was fluorescently stained and scanned on an Illumina iScan according to the Infinium HD Assay Methylation Protocol Guide provided by Illumina.

After data acquisition, an initial quality control step, the DNA methylation data was preprocessed and normalized using the Bioconductor packages *minfi* (version 1.32.0) and *wateRmelon* (version 1.30.0)^32,33^. Previous literature has demonstrated that the within-array normalization combination of Noob + BMIQ (β-mixture quantile normalization) improves signal intensities compared to other approaches^34^. Briefly, the function “*preprocessNoob*” (*minfi*) was used to correct for background fluorescence and dye biases within an array. Next, probes and samples with poor quality were identified and removed. Therefore, samples with more than 10% of probes having detection p-values > 1 × 10^–5^ or samples whose intensity distributions demonstrated irregularities were excluded^35^. Furthermore, Illumina had removed 1031 CpG probes when transitioning to their B1 version of the MethylationEPIC v1.0 manifest due to poor performance and additional probes in the transition from their B2 to their B3 version^36^. In addition to probes flagged by Illumina, we also excluded probes with a median detection p-value > 0.05 from the subsequent statistical analysis. Next, we corrected the type II probe bias using the function “*BMIQ*” (*wateRmelon*) to achieve comparable methylation distributions of type I and II probes^32^. All single nucleotide polymorphism (SNP)-CpG interaction and cross-reactive probes were flagged.

### Statistical analysis

We used the mean and standard deviations (SD) to summarize continuous anthropometric measures and percentages for categorical measures. We modeled all the clinical outcomes, except standardized systolic and diastolic blood pressure, with a multiple linear regression model. Based on prior studies, we included race/ethnicity, sex, and years of age in the regression model^6,22^. The model was completed with the inclusion of the BMIz, HOMA-IR, and the cross-product of BMIz and HOMA-IR. We pointed out that standardization of the systolic and diastolic blood pressure measures accounted for sex and age; hence, we excluded sex and age from these two regression models. Because only 3 of the 42 participants were not non-Hispanic white or non-Hispanic African American, we dichotomized race to indicate non-Hispanic white or otherwise. One participant had an extreme BMIz and HOMA-IR value. This participant was also one of the youngest in the cohort; the participant’s leverage*—*a measure of influence an observation has in a regression model—was 0.93 on a scale from 0 to 1.0. Including the participant’s data influenced statistical inferences. Therefore, we excluded this participant’s data from the results below, but note how inferences change when including this participant’s data, as described in the footnote of Table 2. Of primary interest in the regression were the coefficients on BMIz, HOMA-IR, and their cross-product. These coefficients provide quantitative evidence for our aim. When producing estimated regression lines and means from the model, as presented in Table 1, we set the values of race, sex, and age at their respective overall means, and values of BMIz and HOMA-IR at respective group means. Confidence intervals for population regression lines were computed pointwise.

**Table 2:** Regression coefficients (95% confidence intervals) for age- and sex-adjusted BMI (*z*-score), HOMA-IR, and their interaction when regressed on the indicated outcome from *N* = 41 participants.

Outcome	BMI*z*	HOMA-IR	BMI*z* × HOMA-IR	rMSE
Heart rate (bpm)	−3.62 (−9.41, 2.17)	−0.74 (−13.50, 12.01)	1.51 (−3.33, 6.34)	10.05
Diastolic blood pressure *z*-score^a^	0.17 (−0.23, 0.58)	0.28 (−0.58, 1.13)	−0.05 (−0.38, 0.27)	0.78
Systolic blood pressure *z*-score^a^	0.41 (−0.01, 0.82)	0.57 (−0.30, 1.43)	−0.17 (−0.50, 0.16)	0.79
Low density lipoprotein (mmol/L)	0.05 (−0.37, 0.47)	−0.05 (−0.98, 0.87)	0.06 (−0.29, 0.41)	0.73
High density lipoprotein (mmol/L)	−0.04 (−0.20, 0.11)	−0.05 (−0.39, 0.29)	0.00 (−0.13, 0.13)	0.27
Triglycerides (mmol/L)^a^	0.21 (−0.01, 0.42)	0.04 (−0.43, 0.52)	−0.01 (−0.19, 0.17)	0.37
Total cholesterol (mmol/L)	−0.08 (−0.53, 0.38)	−0.26 (−1.26, 0.75)	0.12 (−0.26, 0.51)	0.79
Glycerol (µmol/L)	−3.87 (−23.06, 15.32)	−32.80 (−75.06, 9.47)	12.62 (−3.39, 28.64)	33.29
Lactate (mmol/L)^a^	0.10 (−0.19, 0.39)	0.42 (−0.22, 1.06)	−0.10 (−0.34, 0.14)	0.50
NEFA (mmol/L)	−0.01 (−0.03, 0.02)	−0.04 (−0.10, 0.02)	0.01 (−0.01, 0.03)	0.04

All regression models also included age, sex, and an indicator of white/non-Hispanic race. The root mean square error (rMSE) is the estimated standard deviation of the outcome after controlling for the aforementioned factors.

*NEFA *non-esterified fatty acids.

^a^When including the extreme participant, for a total of *N* = 42, the following regression coefficients were significantly different from 0: HOMA-IR for diastolic blood pressure *z*-score; BMIz and HOMA-IR for systolic blood pressure *z*-score; BMIz for triglycerides; and HOMA-IR for lactate. Directions of these coefficients were in the same direction as in this table. Magnitudes of these coefficients when including the extreme participant were between 77 and 105% of the corresponding coefficients when excluding the extreme participant for all except the HOMA-IR coefficient for diastolic blood pressure *z*-score, which was 150%.

DNA methylation data were analyzed with the same regression model described above, having the BMIz, HOMA-IR, and their cross-product as the covariates of primary interest, and adjusting for sex, age, as well as race/ethnicity. Analyses were conducted in the R statistical programming language (version 3.6). The statistical analysis for probe-wise differential DNA methylation was performed following the *limma* workflow^37^. *M*-values were calculated by transforming β-values using the logit-transformation ($M=lo{g}_{2}(\frac{\beta }{1-\beta } )$). To evaluate the effects of BMIz, HOMA-IR, and their cross-product on DNAm, we fitted the *M*-values with the same regression model as for the clinical outcomes described above. Again, our inferential interests were on the coefficients on BMIz, HOMA-IR, and their cross-product. We used the “*lmFit*” and “*eBayes*” functions in the *limma* package (version 3.42.2) when fitting the regression models ^37^. For each of the BMIz, HOMA-IR, and their cross-product, *p*-values were adjusted using the Benjamini–Hochberg method^38^ to control the False Discovery Rate (FDR). A CpG was considered differentially methylated if the FDR was < 0.05 for a given effect (BMIz, HOMA-IR, or their cross-product).

## Results

### Anthropometric characterization

From the anthropometric characterization of our cohort, among overweight participants, those who were insulin sensitive (O-IS) had BMIz (*z* = 1.76) that, on average, were more than 1¼ SDs lower than their insulin resistant peers (O-IR) (*z* = 2.36); Table 1. This difference in BMIz and the absence of a normal weight and insulin resistant group (i.e., no N-IR group) speak to the related nature of body mass and insulin sensitivity. The correlation between BMIz and HOMA-IR, across the whole sample of 41 study participants, was 0.57 (95% CI: 0.31, 0.74). Regarding HOMA-IR, by definition, the groups’ HOMA-IR differed. These HOMA-IR differences were largely driven by differences in fasting insulin levels rather than in glucose, as noted by the increased levels of fasting insulin on O-IR group when compared to the O-IS group, while glucose levels remain similar among groups (Table 1).

From the regression analyses results, there were no relationships between the clinical outcomes and the BMIz, or HOMA-IR, or their interaction; all the confidence intervals covered the null value of 0, showing no statistically significant coefficient (Table 2). However, when comparing among the three groups, the regression-estimated means differed on diastolic and systolic blood pressure, *z*-scores, triglycerides and lactate levels, by at least 1 SD. These differences were corroborated with statistical evidence (Table 1). The estimated SDs were the root mean square error (rMSE) from the regression and are presented in Table 2. These differences are described in more depth below.

**Figure 1:**
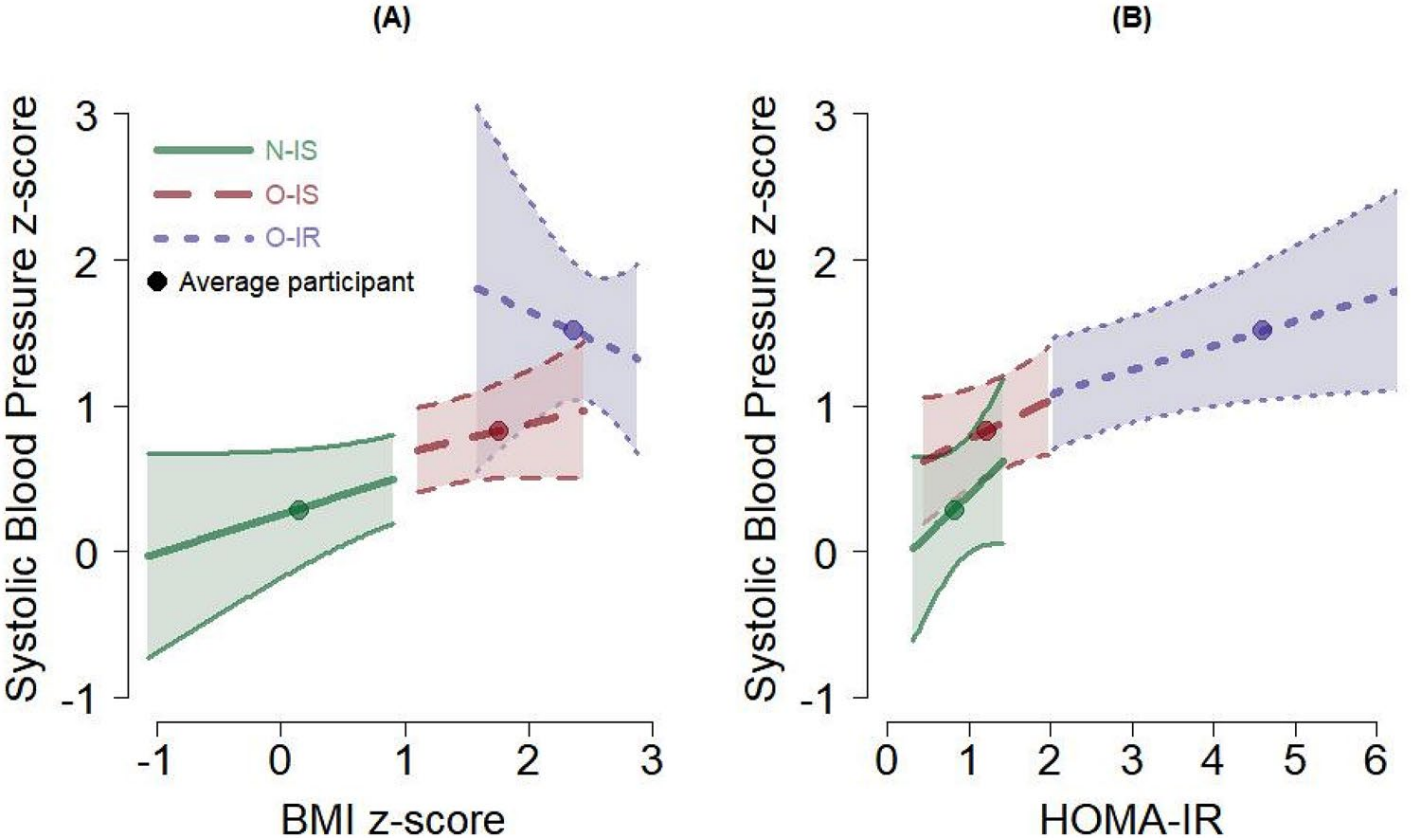
Expected values and 95% confidence intervals of systolic blood pressure (mmHg) from the regression model, profiled by the BMI *z*-score (**A**) and the HOMA-IR (**B**). The expected value for the average participant in each group is also plotted; see Table 1. The segment for each group covers the range of values observed for the given group (except for the O-IR group on the HOMA-IR where the maximum observed HOMA-IR was 9.6). Forty-one participants contributed data for these figures.

Importantly, in regard to blood pressure measurements, higher HOMA-IR values were related to higher blood pressure, while higher BMI values were not. This was evidenced by the blood pressure means from the O-IR group that were much greater than those from the other two groups (Table 1). This is also observed by the estimated regression coefficients for HOMA-IR compared to those for BMI, though these coefficients were not statistically different from 0 (Table 2, Fig. 1 for systolic blood pressure as an example). With the SDs of the standardized (z-score) systolic and diastolic blood pressure measures around 0.80 (Table 2), the O-IR group means (1.52) were about ¾ of an SD greater than the O-IS group mean (0.83), and over 1 SD greater than the N-IS group mean (0.29). A potential interaction of BMI and HOMA-IR is shown in Fig. 1A. Figure 1A shows standardized systolic blood pressure plotted by BMIz, adjusted for HOMA-IR. Here the slope for the O-IR group decreases as BMI increases, whereas for the other two groups, the slopes increase. This different pattern among groups is indicative of an interaction between BMI and HOMA-IR on systolic blood pressure, and when including the participant with extreme BMI, as described above, this interaction was statistically significant. However, with at most 42 observations, we cannot have confidence that BMIz and HOMA-IR work together to influence blood pressure.

**Figure 2:**
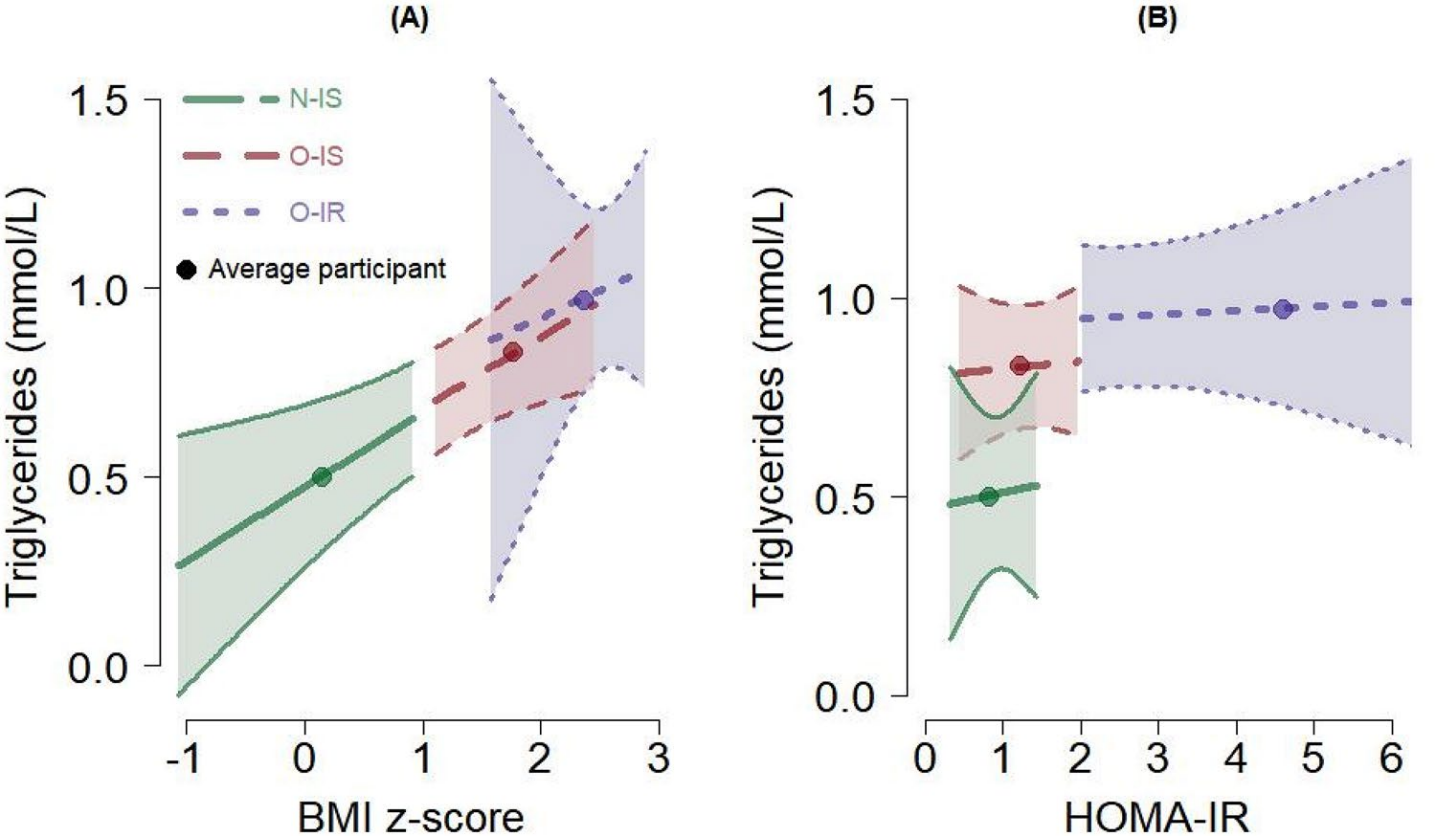
Expected values and 95% confidence intervals of triglycerides (mmol/L) from the regression model, profiled by the BMI z-score (**A**) and the HOMA-IR (**B**). The expected value for the average participant in each group is also plotted; see Table 1. The segment for each group covers the range of values observed for the given group (except for the O-IR group on HOMA-IR where the maximum observed HOMA-IR was 9.6). Forty-one participants contributed data for these figures.

Interestingly, triglyceride levels increased with increased BMI levels. However, triglyceride levels had little to no relationship with HOMA-IR. With the SD of triglycerides at 0.37 mmol/L (Table 2), the N-IS group’s mean (0.50 mmol/L) was 0.86 SD lower than the O-IS’s mean (0.82 mmol/L) and 1.27 SD lower than the O-IR’s mean (0.97 mmol/L) (Table 1). Though the confidence intervals on regression coefficients for BMIz and HOMA-IR included 0 (not significant) (Table 2), the relationships are visually depicted in Fig. 2. The interaction of BMI and HOMA-IR was negligible (Table 2); this can be seen as the slopes for the three groups are similar for BMI (Fig. 2A) and HOMA-IR (Fig. 2B).

**Figure 3:**
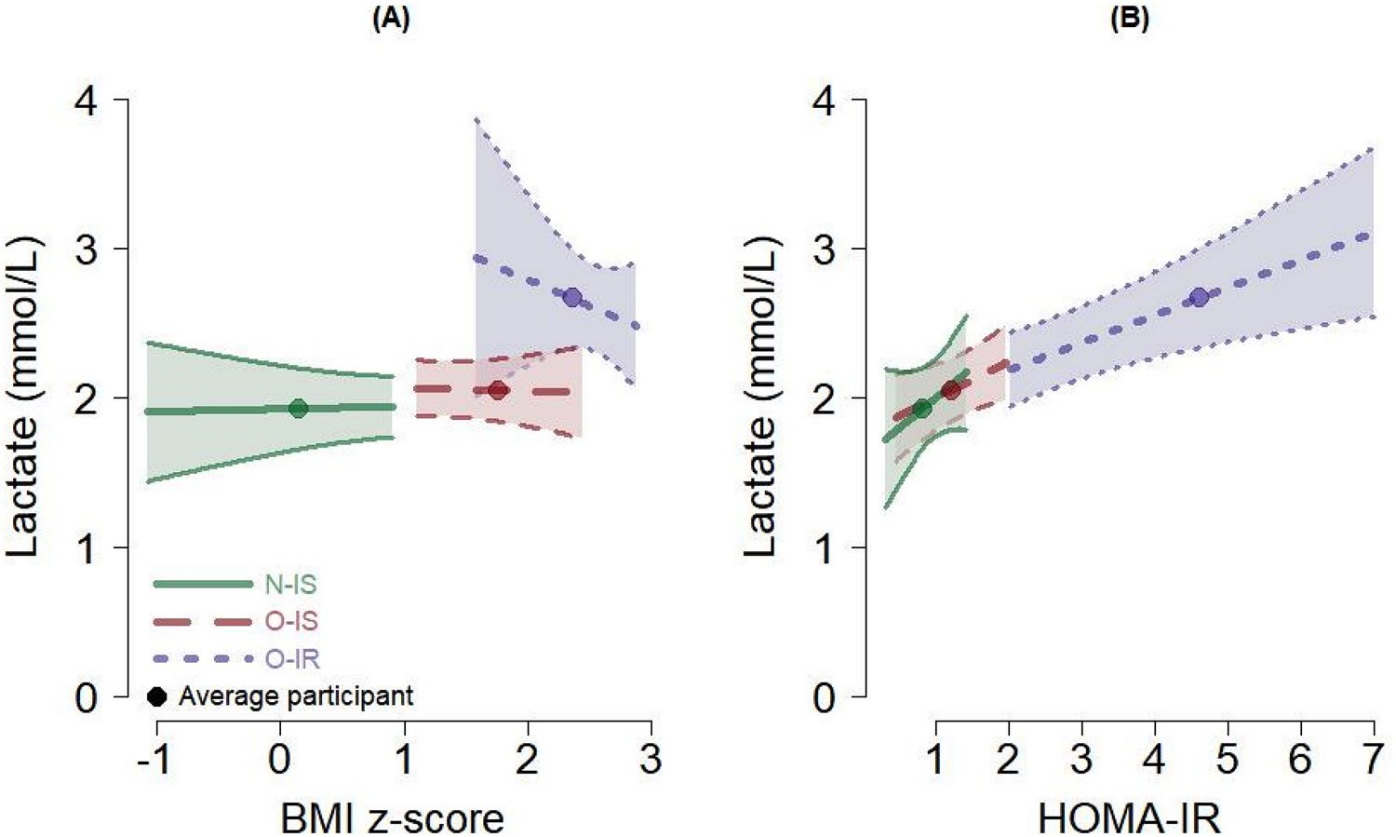
Expected values and 95% confidence intervals of lactate (mmol/L) from the regression model, profiled by the BMI z-score (**A**) and HOMA-IR (**B**). The expected value for the average participant in each group is also plotted; see Table 1. The segment for each group covers the range of values observed for the given group (except for the O-IR group on the HOMA-IR where the maximum observed HOMA-IR was 9.6). Forty-one participants contributed data for these figures.

On the other hand, lactate levels were higher in those with higher HOMA-IR but had little to no relationship with BMIz values. The SD of lactate was 0.50 mmol/L (Table 2). The O-IR group’s mean lactate (2.67 mmol/L) was about 1 SD greater than that for the O-IS group (2.06 mmol/L) and about 1.5 SD greater than that for the N-IS group (1.94 mmol/L) (Table 1). Though the confidence interval for the HOMA-IR coefficient included 0 (Table 2), the positive relationship of lactate to HOMA-IR (adjusted for BMI) is visualized in Fig. 3B. Figure 3A shows the relationship of lactate, or lack thereof, to BMI (adjusted for HOMA-IR). The evidence that BMIz and HOMA-IR work together in their relationship to lactate was also negligible (Table 2, Fig. 3).

In general, BMI and the HOMA-IR seems to have different contribution to the variation of each clinical outcome, affecting clinical outcomes independently. Although, their interaction could be found to affect the blood pressure.

### Genome-wide DNA methylation profiling

The same statistical approach was applied for the DNA methylation analysis and the multiple linear regression model was used as previously described for anthropometric and clinical parameters. We controlled for age, sex and race, as these are important factors that could lead to important changes in the DNA methylation patterns of an individual, as previously reported^6,22^. This way we were able to reduce the bias caused by these types of variables in our results, therefore, focusing only on the specific effects caused by the BMIz, HOMA-IR, or both together.

**Figure 4:**
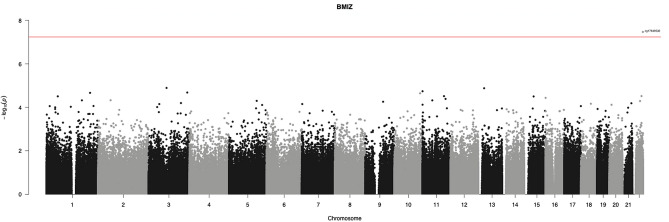
Manhattan plot representing the differentially methylated positions associated with BMI *z-*score and respective chromosomal distribution. The red horizontal line represents the threshold of FDR < 0.05. The Manhattan plot was performed using R package *qqman* (version 0.1.4)^41^. Forty-one participants contributed data for this figure.

Using this statistical approach, only one CpG position (cg17649532) was differentially methylated, presenting an FDR < 0.05, as shown in Fig. 4. This alteration in methylation was exclusively influenced by the BMIz. No statistical differences in association with the HOMA-IR or the cross-product of BMIz and HOMA-IR were found with a threshold of FDR < 0.05. Even when increasing the threshold to FDR < 0.1, no other CpG appeared to be associated with our variables. In Fig. 4, a Manhattan plot is represented, highlighting the CpG position associated exclusively with the BMIz at a threshold of FDR < 0.05. This CpG is located on the phosphatase 6 regulatory subunit 2 (*PPP6R2*) gene in chromosome 22. Mean methylation levels of this CpG were 3.58 (CI95%: 3.48, 3.68) for N-IS participants, 4.00 (CI95%: 3.93, 4.08) for O-IS participants, and 4.03 (CI95%: 3.90, 4.15) for O-IR participants. The mean level for N-IS participants was statistically lower than that for both O-IS and O-IR participants. Interestingly, the difference in means between O-IS and O-IR participants was very small—by 0.02 (CI95%: −0.13, 0.17)—corroborating that BMIz by itself is associated with alterations of the methylation pattern of this DMP. Our data also showed a predictive increase of 0.41 units on CpG methylation. Furthermore, we matched the non-statistically significant CpGs with CpGs already described in the literature. With this analysis we found that 71 CpGs matched with already described CpGs on meta-analysis by Do et al., in people of 18–75 years of age^39^. From these 71, 32 of these CpGs followed the same directionality in terms of methylation patterns, in relation to BMI. Furthermore, Arpón et al. found three CpGs related to HOMA-IR in people (27–57 years). Interestingly, we have found that two out of these three CpGs follows the same direction^40^ (Supplementary Table S1). These associations between the methylation and BMI or HOMA-IR, could indicate that our cohort already presents a similar methylation pattern as it is found in older people. These could lead to an early onset of different comorbidities early in age.

## Discussion

Using a single statistical model, the present study estimated the independent and combined effects of obesity and insulin resistance on clinical outcomes and on PBMC DNA methylation patterns, in prepubertal children with obesity. Our results showed that O-IR children tended to have higher systolic and diastolic blood pressure than their insulin sensitive counterparts and N-IS children, and showed a progressive increase in triglyceride levels. Interestingly, we found a relationship between the HOMA-IR and lactate levels. Higher levels of lactate were present in children with insulin resistance. Moreover, we found an important positive association between BMIz and the hypermethylation of a CpG located in the *PPP62* gene, in children.

The statistical model employed in the current study reduced the bias compared to modelling the effects of obesity and insulin resistance separately. In addition, it allowed for a potential additive or synergistic effect of obesity and insulin resistance on metabolic health to be studied. Further, obesity and insulin resistance are on *a continuum*, rather than simply dichotomous variables, which is how many studies treat these factors ^42–44^. Very few similar studies have used regression. We found no studies where BMI- or insulin-based variables were used as independent variables. Treating these factors as continuous provides more precise interpretability of the data as if treated as dichotomous factors. For example, with results from our regression models, comparisons among insulin sensitive children with BMIz of 1.04 (overweight), 1.64 (obese), and 2.05 (severely obese) are possible, rather than having to assume they are all the same because they are above the 85^th^ percentile. Our results also give insight into how outcomes change with both obesity and insulin sensitivity. This study, in a prepubescent cohort of children with obesity, is of particular importance to understand the development of pre-chronic metabolic conditions and diseases, such as T2D.

Our results indicate that O-IR had higher BMIz than the O-IS peers. It is already known that increasing BMI brings increased risk of cardiovascular disorders^45^. Interestingly, both the O-IS and O-IR groups showed perturbations of systolic blood pressure (SBP) and diastolic blood pressure (DBP). Despite higher SBP and DBP values, these values tended to decrease with the increasing BMIz in O-IR subjects. This indicates that BMI may not be the sole factor responsible for the observed perturbations in blood pressure, but that the contribution of the insulin resistance index may be even more important. Insulin resistance appears to be driving blood pressure alterations more than the BMIz. Importantly, these alterations in blood pressure early in life may be linked to the development of cardiovascular diseases, later in life^46,47^, and insulin resistance seems to play a key role. Falkner et al., described a positive relationship between the increased BMI percentile and high SBP and DBP in children and adolescents 2–19 years old in clinical-based samples^48^. A study conducted by Song, in adolescents 10–19 years old, with and without overweight, found correlations between high BMI and increased SBP after adjusting the analyses for sex and height^46^. This correlation was significant in children with BMI index > 85th percentile, but even higher in adolescents with BMI > 90th percentile^46^. Other risk factors that could contribute to cardiovascular dysfunction in association with high blood pressure is dyslipidemia^49^. Indeed, dyslipidemia is also related with obesity^50^, and usually follows a pattern of increased LDL^51^ and TGs, as well as a reduction in HDL^52^. In our cohort, we observed that the levels of triglycerides seem to be predicted by BMIz, without the influence of HOMA-IR. Deeb et al. examined 216 overweight or obese children and adolescent, 4–19 years old, and found that 55.3% of the subjects presented dyslipidemia^53^.

Interestingly, in our cohort, fasting glucose was similar among the three groups of children. However, the insulin levels were increased in overweight participants, driving the differences in the HOMA-IR index. Insulin resistance can be present years before any detectable alterations in the circulating glucose^18,53^, and it is an important underlying mechanism in the development of cardiovascular diseases^45,54^ and dyslipidemia^50,52^. This insulin resistant state at an early age is anticipated by the increased insulin production from beta-cells in an attempt to maintain euglycemia, leading to a hyperinsulinemic state^15^. This may consequently also induce the observed increase in fasting plasma lactate levels. This is corroborated by Berhane et al., who showed increased plasma lactate in adults during a hyperinsulinemic euglycemic clamp, a method that mimics the hyperinsulinemic state^55^. Interestingly, they also indicated that increased lactate levels was present before insulin resistance could be clinically detected following the hyperinsulinemic state^55^. Studies have linked hyperglycemia and chronic hyperinsulinemia with pathogenetic mechanisms that are associated with alterations in cellular signaling and specific metabolic pathways, namely glucose and lipid metabolism, as well as the cellular redox state^56^. Furthermore, large clinical trials, such as the ACCORD and the ADVANCE trials have evaluated the impact of reducing fasting glucose levels to reduce diabetes complications. Curiously, they have shown that even after normalizing glycemia the complications associated with diabetes were not equally reduced^57,58^. This indicates that pathogenic mechanisms driving obesity-associated comorbidities could be driven by factors other than hyperglycemia.

Epigenetic alterations, in particular DNA methylation, are central in many human conditions and diseases, including obesity^6^. The most studied groups with obesity are adults, adolescents and newborns^59^. There is a paucity of data regarding the impact of overweight, obesity and/or insulin resistance on DNAm in prepubertal children. Hormonal changes that occurs during puberty is an influencing factor on epigenetic alterations during development^60^. Therefore, our study included only pre-pubertal subjects, to exclude the interference of puberty in the results.

We observed that the BMIz had an impact on DNAm in PBMCs. Genome-wide DNA methylation analysis showed that 1 CpG was directly influenced by the BMIz, but not by the HOMA-IR or by their cross-product. Interestingly, this particular CpG is located near an important regulatory gene that expresses the regulatory subunit 2 of protein phosphatase 6^61^. Alterations in the expression pattern of protein phosphatase 6 enzyme will induce the phosphorylation of IkBε with consequent degradation by the proteosome, leading to activation of nuclear factor (NF)-kB1^62^. NF-kB acts as transcription factor and induces the expression of genes^62^, including activation of the inflammatory response^28,62^. It has already been reported that NF-kB is upregulated in obesity^63^. Our data indicate a possible link for this upregulation to epigenetic factors during obesity. Importantly, the development of systemic inflammation during obesity is well documented, although the origins are still partly unknown^28^. The DNAm alteration observed in this study may be related to the early development of inflammation in obesity. Moreover, two other studies show similar methylation patterns in older subjects^39,40^. The most important differences between these studies and ours are the number of participants enrolled and the age of the participants. Our study participants are 20–30 years younger and are therefore a very important pre-disease cohort. Finding no significance in some of the same CpGs could be due to the lower power of our study. However, we do see a similar directionality in the methylation status of these CpGs, when related to BMI and HOMA-IR.

The present study is limited by the number of participants, the lack of other direct measurements, including fitness levels, nutrient intake, as well as possible environmental factors, including the parents’ smoking habits. Future studies should validate the results obtained through methylation analysis and to measure the gene and protein expression of protein phosphatase 6 and NF-kB, as we did not have enough samples to measure them. Moreover, the identified CpG is located near a single nucleotide polymorphism position (SNP) of *PPP6R2*, rs1361861708, that could have some influence on methylation pattern, although, to the best of our knowledge, no information in the literature refers the impact of that SNP on methylation pattern of the specific CpG.

In summary, this study demonstrated a relationship between obesity and insulin resistance and a link with metabolic health in pre-pubescent children. Further, obesity was related to DNA methylation in circulating immune cells (PBMCs), which may present a novel link between obesity and early development of systemic inflammation in children, even in the presence of normal fasting glycemia.

## Supplementary Information

Supplementary Legends.

Supplementary Table S1.

## Data Availability

All data used during the study are included in this article or are available from the corresponding authors upon reasonable request.
