# Supplementary material for: Effect of excess weight and insulin resistance on DNA methylation in prepubertal children
Source: Sci Rep. 2022 May 19;12:8430. doi: 10.1038/s41598-022-12325-y (PMC9120504; doi:10.1038/s41598-022-12325-y)
Supplement: Supplementary file 1 — Supplementary Legends. [file 41598_2022_12325_MOESM1_ESM.docx]

**Effect of excess weight and insulin resistance on DNA methylation in prepubertal children - Supplementary Table S1**

**Supplementary Table S1 –** CpGs following the same methylation pattern direction in comparison to the already described in the literature, specifically by Arpón et al. (2019) and Do et al. (2021). Reference - References used in order to compare our CpG's; CpG - Cistosine-phosphate-Guanine site; BMIZ.HIR - BMIz and HOMA-IR crossproduct coefficient; BMIZ.HIR.LB - BMIz and HOMA-IR crossproduct 95% lower bound; BMIZ.HIR.UB - BMIz and HOMA-IR crossproduct 95% upper bound; BMIZ.HIR.p - BMIz and HOMA-IR crossproduct p-value; BMIZ.HIR.FDR - BMIz and HOMA-IR crossproduct false discovery rate; BMIZ - BMIz coefficient; BMIZ.LB - BMIz 95% lower bound; BMIZ.UB - BMIz 95% upper bound; BMIZ.p - BMIz p-value; BMIZ.FDR - BMIz false discovery rate; HIR - HOMA-IR coefficient; HIR.LB - HOMA-IR 95% lower bound; HIR.UB - HOMA-IR 95% upper bound; HIR.p - HOMA-IR p-value; HIR.FDR - HOMA-IR false discovery rate; MSE - Mean Square Error; Genes - Annotated genes; Chr – Chromossome;

Pos – Position; Flagged - True - All single nucleotide polymorphism (SNP)-CpG interaction and cross-reactive probe.
